# Comparison of airway management without neuromuscular blockers in laparoscopic gynecological surgery

**DOI:** 10.1097/MD.0000000000024676

**Published:** 2021-02-19

**Authors:** Sule Ozbilgin, Bahar Kuvaki, Hatice Keskin Şimşek, Bahadir Saatli

**Affiliations:** a Department of Anesthesiology and Intensive Care; b Department of Public Health; c Department of Obstetrics and Gynecology, School of Medicine, Dokuz Eylul University, Izmir, Turkey.

**Keywords:** gynecology, laparoscopy, laryngeal mask, neuromuscular blocker

## Abstract

New generation supraglottic airway devices are suitable for airway management in many laparoscopic surgeries. In this study, we evaluated and compared the ventilation parameters of the laryngeal mask airway-supreme (LM-S) and endotracheal tube (ETT) when a neuromuscular blocker (NMB) agent was not used during laparoscopic gynecological surgery. The second outcome was based on the evaluation of the surgical view because it may affect the surgical procedure.

This was a randomized study that enrolled 100 patients between 18 and 65 years old with an ASA I-II classification. Patients were divided into 2 groups: Group ETT and Group LM-S. Standard anesthesia and ventilation protocols were administered to patients in each group. Ventilation parameters [airway peak pressure (Ppeak), mean airway pressure (Pmean), total volume, and oropharyngeal leak pressure] were recorded before, after, and during peritoneal insufflation and before desufflation, as well as after the removal of the airway device. Perioperative surgical view quality and the adequacy of the pneumoperitoneum were also recorded.

The data of 100 patients were included in the statistical analysis. The Ppeak values in Group ETT were significantly higher in the second minute after airway device insertion. The Ppeak and Pmean values in Group ETT were significantly higher before desufflation and after removal of the airway device. No significant differences were found between the groups in terms of adequacy of the pneumoperitoneum or quality of the surgical view.

The results of this study showed that gynecological laparoscopies can be performed without using a NMB. Satisfactory conditions for ventilation and surgery can be achieved while sparing the use of muscle relaxants in both groups despite the Trendelenburg position and the pneumoperitoneum of the patients, which are typical for laparoscopic gynecological surgery. The results are of clinical significance because they show that the use of a muscle relaxant is unnecessary when supraglottic airways are used for these surgical procedures.

## 1 Introduction

Laparascopic surgeries are mostly chosen for gynecological cases and the Trendelenburg position is mandatory. In addition, challenges to laparoscopy in the Trendelenburg position require a cautious approach for supraglottic airway device (SGD) use.^[1–5]^ Because of the elevation of the diaphragm, there is a 30% to 50% decline in thoraco-pulmonary compliance, an increase in airway pressure and mean airway pressure, and a decrease in functional residual capacity, as well as ventilation-perfusion mismatch and intraoperative basal atelectasis.^[6]^ The increase in intraabdominal pressure during SGD use may cause the reflux of gastric contents with the risk of regurgitation or pulmonary aspiration.^[7]^ The effect on the pharyngo-esophageal reflux exhibited no relation with studies showing lower esophageal sphincter tone with the use of supraglottic airway device.^[8,9]^ Evidence from the use of a SGD compared with an endotracheal tube (ETT) indicates that supraglottic devices are good alternatives to ETTs for laparoscopic gynecologic surgeries.^[1,10–12]^ SGDs provide equal conditions for surgery with less hemodynamic stress response during insertion and better postoperative analgesia than ETTs.^[1,13]^ In most of these studies neuromuscular blockers were used.

In addition, another important advantage for SGD use is that there is no need for neuromuscular blocking agent (NMB) during insertion.^[14–17]^ Some studies have shown that it is possible to perform laparoscopic gynecologic surgery without a neuromuscular blocking agent.^[2,10,11]^ Additionally, increased incidence of postoperative pulmonary complications, residual paralysis, recurarization, and need for reversal are well-known disadvantages.^[18]^ If neuromuscular blockers are not required for SGD placement, it seems logical to use this advantage throughout the entire surgery.

In a previous study, we showed that a LMA supreme (LM-S) can be used as safely as an ETT for laparoscopic gynecological surgery when NMB is used.^[1]^ Then, we developed the hypothesis that this operation can be performed when NMB is not used. Therefore, the primary aim of this study was to compare an ETT and LMA-supreme for laparoscopic gynecological surgery without the use of a NMB.

## 2 Methods

The University's Institutional Review Board approval (IRB: 859-GOA) was granted, and written informed consent was obtained from all subjects participating in the trial. Prior to patient enrollment, the trial was registered at clinicaltrials.gov (NCT02125838).

This randomized study included 100 patients who were between 18 and 65 years with an ASA I-II classification and undergoing elective laparoscopic gynecological surgery.

### 2.1 Exclusion criteria

Patients in whom supraglottic airway devices would not be preferred were not included in the study. These patients were those with any neck or upper respiratory airway pathology, those at risk of regurgitation/aspiration of gastric contents (previous upper GIS surgery, known hiatal hernia, gastroesophageal reflux, history of peptic ulcer, full stomach, and/or pregnancy), those with low pulmonary compliance or high airway resistance (chronic lung diseases), obese patients (BMI > 35), sore throat, dysphonia and dysphagia, and cases with a predicted difficult airway or history of having it.

### 2.2 Randomization

The patients were divided into 2 groups: the ETT and LM-S groups. Patients and surgeons performing the operation were not aware of which airway device was used. The patients in the groups were determined by block randomization.

### 2.3 Anesthesia management

Patients received standard monitoring before anesthesia induction. For preoperative sedation, intravenous (IV) 0.02 mg·kg^−1^ midazolam was administered. No prophylactic anti-emetics were given. Patients were preoxygenated with 6 L ·min^−1^ oxygen for 3 minutes through a face mask. After 2 minutes of 0.2 μg·kg ·^−1^·min^−1^ remifentanil and 6 mg·kg ^−1^·h^−1^ propofol infusion, IV 1 to 2 mg·kg^−1^ propofol was administered. Then, patients were ventilated with 6 L·min^−1^ 100% oxygen through a face mask.

Bispectral index monitoring (BIS, ASPECT A-2000 BIS XP monitor) was used in an attempt to standardize the anesthesia depth. BIS values were maintained between 40 and 60 and were maintained at this interval by giving a bolus dose and increasing, or if necessary, decreasing, the propofol infusion by 1 mg·kg^−1^. Anesthesia maintenance was provided by 50% O_2_/air mix with 0.1 to 0.4 μg·kg^−1^·min^−1^ remifentanil and 3 to 9 mg·kg ^−1^·h^−1^ propofol IV infusion.^[3]^

Before the LM-S (The Laryngeal Mask Company Limited, Singapore) was inserted, a water-based K-YTM gel (Johnson & Johnson Ltd, Maidenhead, UK) without local anesthetic was applied to completely cover the LM-S cuff to lubricate the surface in contact with the palate. The LM-S size was chosen according to the manufacturer's recommendation based on body weight (1,4,10). After LM-S insertion, the cuff was inflated to a pressure of 60 cmH2O (cuff pressure manometer, Rusch, Germany).

In the ETT group, a 7.5-sized tube was used, and the ETT cuff was inflated until the leak sound ceased, and it was maintained at 20 cmH_2_O.

Airway management was performed by the same specialist anesthesiologist who had over 5 years of experience in airway management (1). Airway insertion was started with the BIS level below 60. During airway device insertion, if necessary, depending on patient reaction, additional doses of 0.5 mg ·kg^−1^ propofol were administered. Successful insertion of the LM-S or ETT was confirmed by square-shaped waves of end-tidal CO_2_ and visible chest movements. After successful placement of the airway device, it was covered before the surgeon entered the OR. All perioperative and postoperative data were recorded by another anesthesiologist, who did not insert the airway device. The time for the successful placement (duration from the mouth opening to first successful ventilation), the number of attempts, and the ease of placement were recorded. The anesthesiologist in charge of the airway evaluated ease of insertion as easy, difficult, or unsuccessful (alternative airway management).^[19,20]^ Patients in whom airway device insertion was unsuccessful after 3 attempts, that is, patients for whom LM-S insertion or intubation was unsuccessful, were excluded from the study.

The oropharyngeal leak pressure was measured after closing the adjustable pressure-limiting valve with a fresh gas flow of 3 L·min^−1^, noting the airway pressure at equilibrium or when there was an audible leak. To prevent exposure of the lungs to barotrauma, the expiratory valve was opened when the peak inspiratory pressure reached 40 cmH_2_O, and the test was concluded.^[20]^ Oropharyngeal leak pressure was measured before peritoneal insufflation, 10 minutes later and immediately before desufflation and was completed by a researcher blind to the type of airway device inserted.

Positive pressure ventilation was volume-controlled with 2 to 4 L·min^−1^ fresh gas flows, 0.5 fractions of inspired oxygen (FiO2) and 6 to 8 mL·kg^−1^ tidal volume. Positive end expiratuar pressure was not administered, and the ETCO_2_ was held between 35 and 45 mm Hg. CO_2_ insufflation for the laparoscopic intervention was allowed until an intraabdominal pressure of 15 mm Hg was obtained.

Ventilation parameters were evaluated 2 minutes after LM-S or ETT placement (T1), 10 minutes after the Trendelenburg position (after insufflation) (T2), immediately before peritoneal desufflation (T3) and before airway device removal (T4). Ventilation measurements were tidal volume (TV), respiration rate (RR), peripheral oxygen saturation (SpO_2_), end-tidal carbon dioxide pressure (PETCO2), peak airway pressure (Ppeak), mean airway pressure (P mean), and expiration volume per minute (VE). The cuff pressures of the SGD and ETT were also measured at the same time intervals.

Evaluation related to the gastric tube: Immediately after airway device placement, in the LM-S group a 14 Ch gastric tube (Biçakçilar Tibbi Cihazlar Sanayi ve Ticaret A.Ş. İstanbul, Turkey) was inserted through the LM-S drainage tube. In the ETT group the gastric tube was inserted through the oral airway. The person who inserted the gastric tube classified the ease of placement as very easy, easy, difficult, or very difficult.^[5]^ Immediately after the intra-abdominal laparoscopic intervention and immediately prior to the end of peritoneal insufflation, the surgeon blind to the airway device, evaluated gastric distension on a scale of 0 to 10 (0=empty stomach to 10=distension obstructing the surgical field).^[5]^

The number of interventions with Veress needle, initial intra-abdominal pressure, the duration to reach an intra-abdominal pressure of 15 mm Hg and an insufflated volume of CO_2_ were recorded. At the end of the procedure, the surgeon evaluated the quality of the pneumoperitoneum (adequate/inadequate) and graded it on a 4-point scale (1-poor to 4-excellent).^[16]^

After the removal of the LM-S or ETT at the end of surgery, the total duration of anesthesia and peritoneal insufflation were recorded. Possible complications that could develop during airway device removal (coughing, vomiting, laryngeal stridor, laryngeal spasm or requirement for airway intervention) were noted.

After the SGAD was removed, the presence of blood (1 = no blood, 2 = trace amounts of blood, and 3 = clear amounts of blood) on the device was evaluated. A blinded researcher evaluated the laryngopharyngeal symptoms of the patients in the recovery unit in the first hour and then 24 hours later. Sore throat was evaluated with VAS-10 (visual analogue scale), while dysphonia and dysphagia were assessed as yes/no.

### 2.4 Power analysis

In a study that was conducted by Seet et al^[20]^ power was calculated as 90% as a result of the power analysis that was performed for the laryngeal mask airway OLP=>21+/−5 cmH2O average values, and the number of the cases was determined to be at least 40 for each group. Type I alpha error coefficient maximum of 0.05 and type II beta error coefficient maximum of 0.2 were determined.

### 2.5 Statistical analysis

To determine whether the difference between the measurements in the 2 groups were significant, *t* tests were used for the independent groups, while chi-square tests were used for grouped data. To determine whether the differences in measurements at different times in the same groups were significant, repeated ANOVA *t* was used. When the difference was significant, Bonferroni's corrected *t* test was used to determine which group caused the difference. The significance level for Bonferroni's corrected *t* test was 0.05, which was taken as a comparison count. The significance level for all tests, except Bonferroni's corrected *t* test, was *P* < 0.05.

## 3 Results

**Figure 1:**
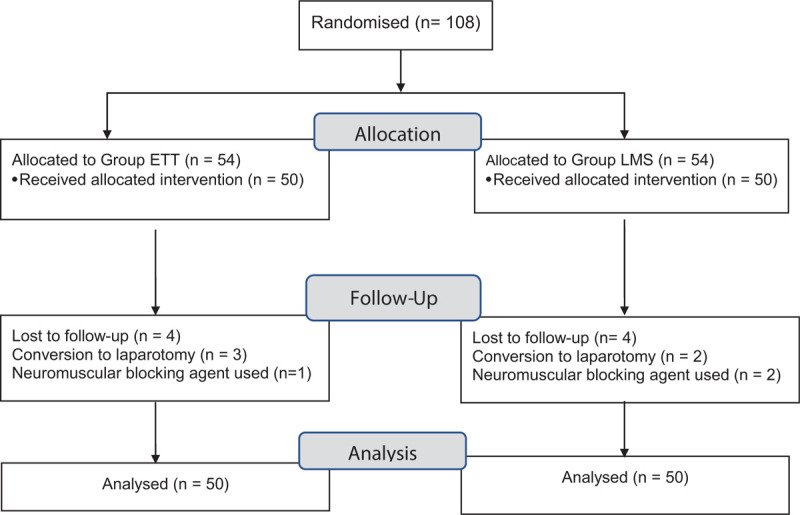
Flow chart.

The study was initiated with 108 patients. However, only 100 patients could be included in the statistical analysis (Fig. 1). The 2 study groups were comparable in terms of demographic characteristics (Table 1). No ventilation failures occurred with either airway. A total of 8 patients were excluded from the study. For 3 patients, the scheduled procedure was changed from laparoscopic to open surgery based on surgical reasons in Group ETT. For 1 patient in Group ETT, a neuromuscular blocking agent used. Two patients with a scheduled laparoscopic procedure required open surgery for surgery-related reasons and were excluded from Group LM-S. For 2 patients in Group LM-S, a neuromuscular blocking agent was used.

**Table 1:** Demographic characteristics of the patients.

Group	ETT (n = 50)	LM-S (n = 50)	*P* value
Age; years	37 ± 11	38 ± 11	0.374
Body mass index; kg·m^−2^	26 ± 10	23 ± 5	0.055
ASA class 1/2	31/19	36/14	0.288
Mallampati class 1 / 2	28/22	35/15	0.704
Airway insertion time; s	22 ± 9	14 ± 3	<0.001^∗^
Number of insertion attempts	1.32 ± 0.71	1.02 ± 0.14	= 0.005
Ease of insertion of the airway device			
Easy	41 (82%)	50 (100%)	<0.001^∗^
Difficult	9 (18%)	0 (0%)	
Anesthesia duration (min)	121.88 ± 55.69	118.34 ± 68.46	0.779
Insufflation time; minutes	81.46 ± 41.86	79.89 ± 53.98	0.872
Type of surgery			
Total laparoscopic hysterectomy	14 (28%)	16 (32%)	0.413
Laparoscopic cystectomy	23 (46%)	21 (42%)	
Laparoscopic myomectomy	6 (12%)	21 (42%)	
Diagnostic laparoscopy	3 (6%)	7 (14%)	
Laparoscopic tubal ligation	2 (4%)	0 (0%)	
Others	2 (4%)	3 (6%)	
Ease of gastric tube insertion			
Very Easy	19 (38%)	38 (76%)	0.001^∗^
Easy	23 (46%)	10 (20%)	
Difficult	7 (14%)	2 (4%)	
Very difficult	1 (2%)	0 (0%)	

When compared with Group LM-S, the insertion time was significantly longer in Group ETT (21.62 ± 9.0 s) than in Group LM-S (13.86 ± 2.88 s) (*P* < 0.001) (Table 1). There was a significant difference between the groups in the ease of the placement of the gastric tube (*P* < 0.001). There was no significant difference in gastric distension immediately after the laparoscope entered the abdomen (*P* = 0.679) and immediately before desufflation (*P* = 0.716).

The ventilation data were compared between the groups at T1, T2, T3, and T4, and the results are presented in Table 2. There was a significant difference in oropharyngeal leak pressure (OLP) between Group ETT and Group LM-S (*P* < 0.001). The average pressure in Group ETT was 39.5 cmH_2_O, while for Group LM-S, the average was 25.60 ± 4.93 cmH_2_O. There were no significant differences in the OLP values in any of the time periods (T1 to T4) for the LM-S group (F = 0.90, *P* = 0.448) and Group ETT (*P* > 0.05).

**Table 2:** Comparison of the ventilation parameters between groups.

	2 minutes after airway device insertion		10 minutes after insufflation		Before desufflation		Before removal of airway device	
	T1		T2		T3		T4	
Group	ETT	LM-S	ETT	LM-S	ETT	LM-S	ETT	LM-S
Tidal volume (mL)	452 ± 63 (300–620)	437 ± 62 (340–650)	457 ± 57 (350–600)	444 ± 62 (305–650)	462 ± 63 (310–620)	451 ± 59 (340–650)	465 ± 59 (340–630)	448 ± 54 (340–650)
Expirium volume (L·dk^−1^)	5.4 ± 2 (2–13)	5 ± 1 (3–8)	5.8 ± 1.6 (3–13)	6 ± 1 (4–9)	6 ± 2 (4–13)	6 ± 1 (5–9)	6 ± 2 (4–13)	6 ± 1 (4–10)
ETCO_2_ (mm Hg)	32 ± 3 (25–42)	33 ± 1 (24–41)	34 ± 3 (28–43)	^∗^ 35 ± 3 (28–42)	34 ± 3 (28–46)	35 ± 3 (30–46)	33 ± 3 (24–40)	33 ± 3 (27–41)
Peak airway pressure (cmH_2_0)	^∗^ 13 ± 4 (6–24)	12 ± 3 (8–22)	20 ± 5 (8–30)	20 ± 5 (8–29)	19 ± 6 (8–34)	18 ± 4 (8–30)	^∗^ 15 ± 3 (7–22)	13 ± 3 (8–23)
Mean airway pressure (cmH_2_0)	6 ± 2 (4–13)	6 ± 2 (4–13)	9 ± 2 (4–15)	8 ± 3 (4–28)	8 ± 2 (5–13)	8 ± 2 (5–11)	^∗^ 7 ± 1 (5–11)	6 ± 1 (4–9)

Comparing Group ETT and Group LM-S, the peak airway pressure in Group ETT was found to be significantly higher in the second minute after airway device insertion T1 (*P* < 0.001) (Table 2). The peak airway pressure (*P* = 0.041) and mean airway pressure in Group ETT were found to be significantly higher than that in Group LM-S at T3 and T4 (*P* = 0.032).

In terms of the Veress needle entry attempts, initial intraabdominal pressure, time to reach constant pressure of 15 mm Hg, volume of insufflated CO_2_, adequacy of the pneumoperitoneum, and quality of view, there were no significant differences between the groups (Table 3).

**Table 3:** Surgical and insufflation data.

Group	ETT (n = 50)	LM-S (n = 50)	*P* value
Number of Verres needle insertions			
1	47	46	0.432
2	2	4	
4	1	0	
Initial intraabdominal pressure (mm Hg)	3.53 ± 2.28	3.60 ± 2.10	0.297
Time to reach constant 15 mm Hg (s)	115.0 ± 65.4	126.3 ± 54.8	0.352
Volume of insufflated CO_2_ (L)	3.57 ± 1.77	3.98 ± 2.80	0.388
Adequacy of the pneumoperitoneum			
Adequate	50 (100%)	50 (100%)	
Inadequate	0 (0%)	0 (0%)	
Grade of quality of view			
1	0	0	0.827
2	2 (4%)	1 (2%)	
3	10 (20%)	11 (22%)	
4	38 (76%)	38 (76%)	

In the postoperative first hour, sore throat (*P* < 0.001), dysphonia (*P* < 0.001), and dysphagia (*P* < 0.021) were significantly higher in Group ETT. After 24 hours, there was no difference in sore throat, dysphonia, or dysphagia in the 2 groups (Table 4).

**Table 4:** Evaluation of pharyngolaryngeal discomfort.

Group	ETT (n = 50)	LM-S (n = 50)	*P* value
Sore throat			
1. hour	3 ± 2	1 ± 1	<0.001^∗^
24. hour	0 ± 0	0 ± 1	= 0.106
Dysphonia			
1. hour (yes/no)	9/41	2/48	= 0.026^∗^
24. hour (yes/no)	0/50	0/50	
Dysphagia			
1. hour (yes/no)	16/34	0/50	= 0.006^∗^
24. hour (yes/no)	0/50	1/49	= 0.500

The heart rate values were similar in both groups. The systolic blood pressure in Group ETT was found to be significantly higher in the T2, T3, and T4 periods than it was in Group LM-S (*P* < 0.019, *P* < 0.093, and *P* < 0.020, respectively). The diastolic blood pressure in Group ETT was found to be significantly higher in the T2, T3, and T4 periods than it was in Group LM-S (*P* < 0.034, *P* < 0.021, *P* < 0.004, respectively). Compared to that in Group LM-S, the mean blood pressure in Group ETT was found to be significantly higher in the T2, T3, and T4 periods (*P* < 0.014, *P* < 0.0173, and *P* < 0.003, respectively).

## 4 Discussion

Satisfactory conditions for ventilation and surgery can be achieved with LM-S and ETT during laparoscopic gynecologic surgery in the Trendelenburg position when NMB was used.^[4,10,13,19,20]^ In this study, however, we found that good surgical and ventilation conditions could also be achieved when NMB was not used.

Williams et al^[16]^ compared groups with NMB/ETT and without NMB/LM and found no difference in volumes of CO_2_ insufflation or time to reach the desired pneumoperitoneum for laparoscopy gynecological surgery. They emphasized that the total duration of anesthesia was shorter in the LMA group without NMB. In our study, there was also no difference in CO_2_ insufflation volume or time to reach the pneumoperitoneum in either group. In addition, Miller et al^[10]^ concluded that SGDs were superior for day-case laparoscopic surgery, because there was no need for NMB and the duration of stay in the OR was shorter.^[2]^ Neuromuscular blocker agents were not used during induction or surgery in our study, and the operations were performed by the same surgeon. The surgical view quality was rated as 4 (the best) for 76% of the 2 groups. The pneumoperitoneum sufficiency was found to be 100% for both groups, with no patient assessed as having insufficient levels. The number of Veress needle entry attempts was 1 for 47 patients in the Group ETT and for 46 patients in the LM-S group.

Chassard et al^[21]^ compared the use of atracurium and no muscle relaxant for gynecologic laparoscopic surgery under TIVA and found no difference in surgical conditions between the groups. The use of muscle relaxants had no effect on the hemodynamic or ventilatory parameters or the surgical conditions during the pneumoperitoneum for gynecologic laparoscopy in most cases. At a proper depth of anesthesia, spontaneous breathing can be easily inhibited by strong opioids and sedative drugs.

In a study where ProSeal was used for gynecologic laparoscopy in 120 patients, Chen et al^[11]^ showed that there were similar ventilation and surgical conditions when muscle relaxant was not used. They stated that the use of muscle relaxant led to prolongation of surgery and recovery, and therefore, its use had no benefit.^[11,22]^

The average insertion times in this study have been proven in similar studies in which both airway devices were used (14 vs. 22 s).^[23–25]^ In addition, the first-attempt insertion rate was higher with the LM-S than the ETT, similar to other studies with or without NMB.^[1,24,26–29]^ The reason for this outcome may be the need for more time to do laryngoscopy for the insertion of the ETT instead of when only an SGD is inserted or because of lack of a neuromuscular block, which may make endotracheal intubation more difficult. In the study, 6 patients needed additional propofol doses during ETT insertion. The aim of our study was not to compare airway management conditions; therefore, we were aware that we might need more hypnotics during ETT. The aim of our study was to see whether we could avoid using NMB during the whole surgery when SGD is used because no NMB is needed for its insertion.

To avoid gastric distention, insertion of a gastric tube is routine in laparoscopic surgery. We inserted the gastric tube very easily in 76% and easily in 20% of the patients in the Group LM-S and in 38% and 46% of the patients in the Group ETT, respectively. The difference was significant. This low percentage of easy insertions of the gastric tube in the Group ETT had surprised us in our previous study.^[1]^ For the ETT, the success rate for the first attempt with the gastric tube varied between 66% and 80%.^[30,31]^ The first attempt success at inserting a gastric tube through the gastric channel of the LM-S, however, was reported to be 97% to 100%.^[3,16,20,27,30,31,34]^ The gastric tube of the LM-S cannot be placed incorrectly and directly creates the correct route to the esophagus.^[27]^ In the ETT group, the gastric tube can fold or can be directed to different areas within the mouth, possibly making insertion into the esophagus difficult. Additionally, compared with the Group LM-S, the difficult insertion of the gastric tube in the Group ETT may have led to higher pharyngolaryngeal morbidity.^[1,4]^ In some studies on the use of the classic laryngeal mask airway (LM-K), ProSeal laryngeal mask (LM-P), LM-S, and ETT for laparoscopic surgeries, there was no significant difference between the gastric distension scores, which is similar to our results.^[1,4,32]^

Oropharyngeal leak pressure (OLP) is widely used to assess the airway seal of the SGD and is an important marker of the degree of protection of the airway and success of positive pressure ventilation. In our study, the mean OLP for Group LM-S was 26 cmH_2_O, and this result coincides with values reported by other studies using LM-S for laparoscopic gynecological surgeries.^[1,20,33]^ Each patient in our study could be ventilated sufficiently. The Ppeak values in the Group ETT were found to be significantly higher 2 minutes after the airway device was inserted and immediately prior to the removal of the ETT. This result may indicate that there is more airway reaction to an infraglottic airway device such as the ETT than to a supraglottic device. Pmean values in our study were increased upon removal of the ETT. These findings are similar to other studies. In their previous study, Kuvaki et al^[1]^ evaluated the same parameters and surgeries as in this study in LMA-S and ETT groups. Compared to the present study, before and after the pneumoperitoneum in the LM-S group the airway reaction was higher at Ppeak even though they used neuromuscular blockers. These data show that the absence of NMB in laparoscopic gynecologic surgeries has no negative effects on both peak or mean airway pressures.

While pharyngolaryngeal complications are reported between 0% and 20% for LM-S,^[3,4,19,20,27,34,35]^ for ETT, this rate varies from 0% to 47%.^[4,24,33]^ In these studies, LM-S cuffs were held at 60 cmH_2_O or lower or were inflated until the leak sound ceased or pressures were not measured. In our study, cuff pressures were maintained at 60 cmH_2_O for LM-S and at 20 cmH_2_O for the ETT, as recommended for cuff inflation pressures of these airway devices. Upon evaluation in the first hour and 24th hour, pharyngolaryngeal complications were found to be higher in the ETT Group. These results are in accordance with the literature.^[3,4,19,20,33–35]^ In the LM-S group, 1 patient reported dysphagia during the 24th hour evaluation.

We had to use NMB agents in 3 patients in both groups. One patient in Group ETT received a neuromuscular blocking agent. An article by Fülesdi emphasized that the use of a NMB for laparoscopic procedures is a complicated concept.^[36]^ The concept of the use of deep NMB agents during laparoscopic procedures is based on the assumption of keeping the surgical field view optimal while allowing low intraabdominal pressure to be administered. Even without the administration of neuromuscular blocker agents, there are reports showing their applicability for short gynecological laparoscopic procedures.^[11,20,35]^ Our results also support other studies in the literature on the ability to perform laparoscopic surgery without using NMB.^[11,20,24,35]^

This study has a few limitations: This study obtained results in a single center with a small sample. We used only 1 type of LMA. We excluded patients with expected difficulty in airway management or BMI > 35. We did not record the total consumption of propofol and remifentanil.

The conclusion of this study is that gynecological laparoscopies can be performed without using NMB. Satisfactory conditions for ventilation and surgery can be achieved while sparing muscle relaxants in both groups despite the Trendelenburg position and the pneumoperitoneum, which is the routine for laparoscopic gynecological surgery. The results are of clinical significance because they indicate that the use of muscle relaxants is unnecessary when supraglottic airways are used for these surgical procedures.

## Author contributions

**Conceptualization:** Sule Ozbilgin.

**Data curation:** Sule Ozbilgin, Hatice Şimşek Keskin, Bahadir Saatli.

**Formal analysis:** Hatice Şimşek Keskin.

**Investigation:** Sule Ozbilgin, Bahar Kuvaki, Bahadir Saatli.

**Methodology:** Bahar Kuvaki, Sule Ozbilgin.

**Resources:** Bahar Kuvaki, Bahadir Saatli.

**Supervision:** Bahar Kuvaki.

**Validation:** Bahar Kuvaki, Hatice Şimşek Keskin.

**Writing – original draft:** Sule Ozbilgin.

**Writing – review + editing:** Bahar Kuvaki.
